# Non-Animal Hyaluronic Acid and Probiotics Enhance Skin Health via the Gut–Skin Axis: An In Vitro Study on Bioavailability and Cellular Impact

**DOI:** 10.3390/ijms26030897

**Published:** 2025-01-22

**Authors:** Rebecca Galla, Simone Mulè, Sara Ferrari, Claudio Molinari, Francesca Uberti

**Affiliations:** 1 Laboratory of Physiology, Department for Sustainable Development and Ecological Transition, University of Piemonte Orientale, UPO, 13100 Vercelli, Italy; 2 Noivita S.r.l.s., Spin Off of University of Piemonte Orientale, Via Solaroli 17, 28100 Novara, Italy

**Keywords:** hyaluronic acid, probiotic, skin wellness, gut–skin axis, oral supplementation

## Abstract

Hyaluronic acid (HA) represents a pivotal component of the extracellular matrix, particularly within the context of the skin. The absorption and metabolism of orally ingested HA have been extensively investigated due to the prevalence of HA-based supplements. The objective of this study was to evaluate the impact of a combination of non-animal HA and *Bifidobacterium longum* novaBLG1 on dermal health following intestinal transit. The bioavailability of the compound was evaluated using a model that reproduced the human intestinal barrier in vitro, and its biological effects were investigated on skin cells via the gut–skin axis. The results demonstrated that probiotics augmented the absorption of non-animal HA by approximately 30% in comparison to non-animal HA alone and by 82% in comparison to sodium hyaluronate. Furthermore, the combination demonstrated a notable enhancement in skin cell proliferation, with increases of 16%, 8%, and 29.7% over 144 h in comparison to non-animal hyaluronan, *Bifidobacterium longum* novaBLG1, and sodium hyaluronate, respectively. The combination was observed to positively affect all markers of skin health and well-being, achieving its goals without any adverse effects on the gut. This approach offers a novel method for enhancing skin health.

## 1. Introduction

The skin is one of the largest organs in the human body, comprising 16% of body weight and an average surface area of 1.85 m^2^. It maintains homeostasis and acts as a protective barrier against the external environment, preventing infections and fluid loss [1,2]. The skin also plays crucial roles in immune response and as a neuroendocrine organ, with nerves that respond to external stimuli and glands that secrete specific substances. Thus, any injury compromising the skin’s integrity must be quickly repaired to restore its functions [3,4].

Skin ageing results from both intrinsic and extrinsic factors. Intrinsic ageing is genetically driven and involves processes such as epidermal and dermal thinning and increased dryness. Conversely, extrinsic ageing is caused by external factors such as UV radiation or harmful substances like cigarette smoke. This type of ageing is characterised by noticeable wrinkles, reduced elasticity, the thickening of the outer skin layer, dryness, sagging, rough texture, and irregular pigmentation patterns [5]. The most significant age-related alterations manifest predominantly on the facial region, neck, forearm, and backside of the hands [6]. Several factors define skin ageing, including desiccation, a decline in skin firmness, and the occurrence of creases. Moreover, skin ageing has garnered significant interest due to escalating beauty standards. Given the trend towards ageing populations in many nations, the psychosocial impacts of skin ageing amplify the demand for efficacious interventions.

Against this backdrop, nutraceuticals have recently been used as supplementary agents [7]. Hyaluronic acid (HA) is a native constituent extensively prevalent in the extracellular matrix of the human body, predominantly located in the skin. HA showcases remarkable hydrating characteristics and contributes to many physiological mechanisms, encompassing wound healing, tissue restoration and rejuvenation, inflammatory reactions, and embryonic growth [8]. HA assumes a pivotal role within the skin by establishing hydrogen bonds with water molecules, overseeing aquaporin-3 (APQ3) functionality, prompting the migration and proliferation of endothelial cells, and fostering collagen synthesis [9]. Nevertheless, the decrease in HA levels in the skin due to the ageing process leads to a significant reduction, which ultimately culminates in reduced skin elasticity, dryness and wrinkling [10]. Given the widespread use of HA-containing supplements, many scientists have been studying the processes involved in the absorption and metabolism of orally administered HA. It has been found that the degradation of HA in the gastric environment depends on its molecular weight. In particular, high-molecular-weight hyaluronic acid (HMW-HA ≥ 100 kDa) undergoes minimal degradation while crossing the gastric system [11]. After this, HA is absorbed by the intestines’ epithelial cells, M cells, dendritic cells, and macrophages. Upon reaching the lower intestinal epithelial cells [12], HMW-HA interacts with Toll-like receptor 4 (TLR-4) receptors, facilitating uptake. On the other hand, when orally consumed, low-molecular-weight HA (LMW-HA, MW < 100 kDa) is predominantly taken up in the cecum and distributed throughout the body via the circulatory system [13]. In contrast, HMW-HA is mainly transported to the gut-associated lymphoid tissue before being disseminated throughout the body via the bloodstream [14]. In recent years, scientific research has concentrated on elucidating the relationship between oral HA intake and an enhanced dermal condition. This has entailed investigating the impact of LMW-HA on individuals presenting with xerosis. The results of several studies indicate that LMW-HA and HMW-HA are effective in increasing skin hydration levels and reducing the effects of dry skin. One clinical study, which was conducted on patients of different ages who underwent oral administration of HA, yielded the following results: the administration of HMW-HA (300 kDa) at a dosage of 100 mg and 200 mg daily was observed to promote skin hydration after a period of 2–8 weeks, with a significant effect in all treatment groups, which were divided according to age. At the 12-week mark, three additional benefits were observed at the dermal level: increased brightness, diminished yellowness, and augmented epidermal thickness [11,15].

Furthermore, there is a clear association between specific dermatological conditions and certain gastrointestinal diseases. It is therefore unsurprising that the intimate relationship between the gut and the skin manifests more overtly in certain disease states, although the underlying pathobiological basis is often not fully understood [16]. Indeed, several distinct dermatological conditions may indicate a primary underlying gastrointestinal disorder, including inflammatory bowel disease, coeliac disease, and acne rosacea [17,18,19].

Given that the skin and gut are in direct contact with the external environment, healthy ageing is associated with optimal interactions between the gut and skin, underscoring the importance of maintaining physiological equilibrium between these two organs [20]. Ultraviolet radiation represents the principal external factor responsible for the ageing of the skin: ultraviolet radiation stimulates signalling pathways that ultimately increase the transcription of key target genes for photoageing, resulting in increased laxity, dryness, and pigmentation [21,22]. Numerous investigations have sought to elucidate the manner in which the modulation of the gut microbiome may affect immune signalling pathways, thereby mitigating the adverse effects of UV radiation. For instance, lipoteichoic acid (LTA), a component of the Lactobacillus cell wall, is acknowledged for its anti-inflammatory attributes [23]; another investigation indicated that the oral administration of *Lactobacillus plantarum* HY7714 effectively inhibited UV-induced photoageing in murine models by suppressing MMP-1 expression in dermal fibroblasts [24]. In addition, the anti-ageing effect of Lactobacillus plantarum HY7714 has been demonstrated in human studies: in a double-blind, placebo-controlled study, oral supplementation of this probiotic strain in 110 middle-aged subjects resulted in an improvement in skin elasticity and hydration after 12 weeks of treatment [25]. Consequently, an increasing body of evidence from research studies has provided compelling support for the existence of a ‘gut–skin axis’ hypothesis. This has offered a potential avenue for the development of new treatments for a range of dermatological conditions by influencing the balance of the gut microbial community [26]. Among the numerous probiotic strains currently in existence, those belonging to the *Bifidobacterium* family have also been the subject of research into their potential role in maintaining skin health. *Bifidobacterium*, identified as a predominant bacterium within the host gut, is crucial in maintaining host well-being [27]. Existing research has explored the potential of *Bifidobacterium* administration via infusion to improve host health [28]. While limited evidence exists regarding the impact of *Bifidobacterium* on skin homeostasis, recent research indicates that *Bifidobacterium* could potentially function as an anti-ageing agent when incorporated into cosmetic formulations [29]. For example, Hong et al. [30] have demonstrated that bacteria of the Bifidobacterium species are able to cope with UV radiation-induced oxidative damage by restoring cell viability and collagen expression. Results obtained by Gao et al. [31] showed that ageing is capable of altering the composition of the gut microbiota, causing disturbances in the gut–skin axis. However, such damage was reversed after administration with *Bifidobacterium longum*, confirming the anti-ageing effect of this probiotic strain.

Building upon these fundamental premises, the hypothesis that guided this study was to ascertain whether it is possible to utilise non-animal HA in conjunction with a probiotic strain to exert an influence on the full range of physiological mechanisms that are involved in the maintenance of dermal well-being, through the modulation of the gut–skin axis.

## 2. Results

### 2.1. Dose–Response and Time-Course Study of Non-Animal HA and Bifidobacterium longum novaBLG1 in Caco-2 Cells

**Figure 1:**
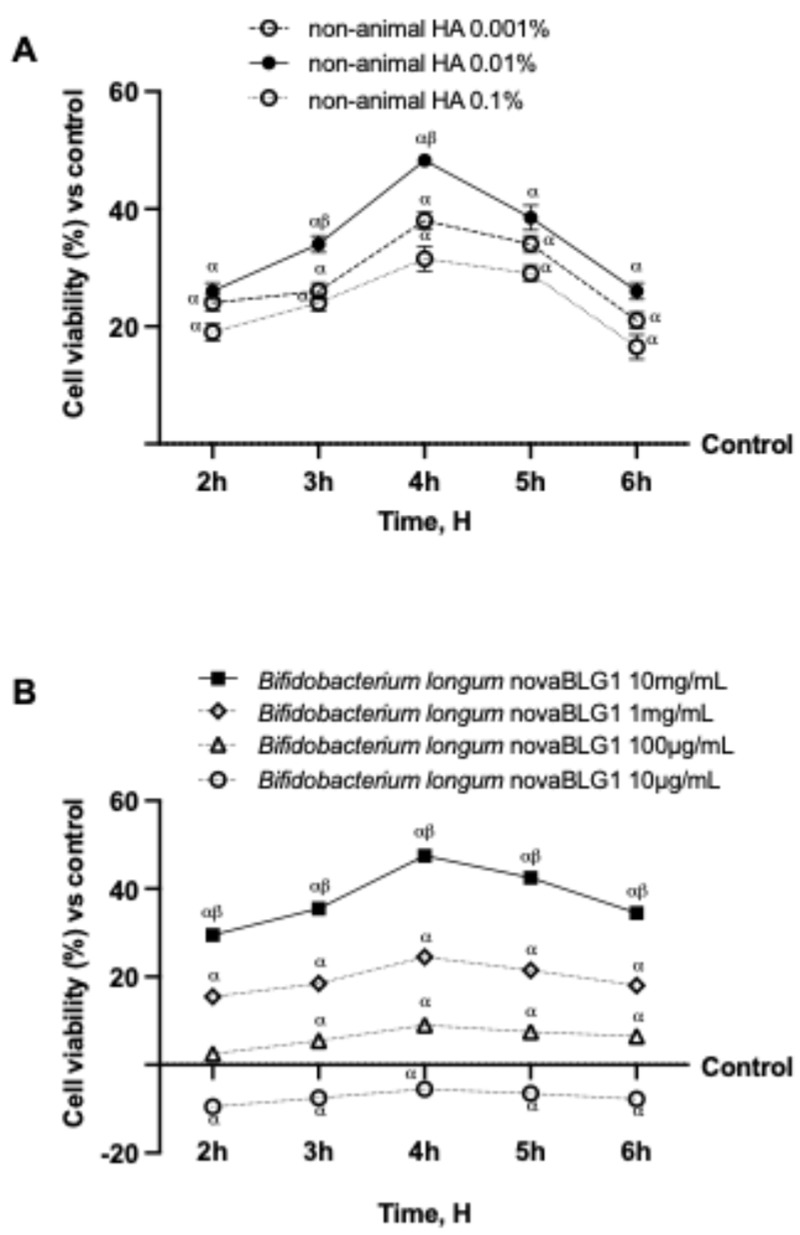
Screening of non-animal HA and probiotic strains in Caco-2 cells by MTT test. (**A**) Cell viability of Caco-2 cells after treatment with different concentrations of HA; (**B**) cell viability of Caco-2 cells after treatment with different concentrations of *Bifidobacterium longum* novaBLG1. Data are expressed as mean ± SD (%) of 5 independent experiments normalised to control (0% line). α *p* < 0.05 vs. control; β *p* < 0.05 vs. other concentrations.

In the first part of the experiments, the intestinal cells Caco-2 were used to perform a screening analysis to choose the best concentrations of non-animal HA (ranging from 0.001% to 0.1%) and *Bifidobacterium longum* novaBLG1 (ranging from 10 µg/mL to 10 mg/mL) to utilise in the second part without adverse effects. For this reason, Caco-2 cells were treated with different concentrations of non-animal HA and *Bifidobacterium longum* novaBLG1, and cell viability was analysed by the MTT test. In Figure 1A, all non-animal HA concentrations show a more significant effect than the control (untreated cells, *p* < 0.05). In particular, non-animal HA 0.01% is the best concentration compared to the other concentrations tested (*p* < 0.05), exhibiting a significant effect at 4 h of treatment of about 53% and 27% compared to the 0.001% and 0.1% concentrations, respectively. As shown in Figure 1B, a concentration screening of the probiotic strain *Bifidobacterium longum* novaBLG1 (ranging from 10 µg/mL to 10 mg/mL, equivalent to 1 × 10^6^–1 × 10^9^ cfu, as illustrated in Figure 1B) was performed. It was found that all tested concentrations, except 10 µg/mL, were able to enhance cell viability throughout the treatment period, with a maximum effect observed at 4 h compared to the control (*p* < 0.05). In particular, the best concentration was found to be 10 mg/mL compared to the others, with the highest effect at 4 h (about 95% vs. 10 µg/mL, 81% vs. 100 µg/mL, and 48% vs. 1 mg/mL, *p* < 0.05). In conclusion, the non-animal HA 0.01% and *Bifidobacterium longum* novaBLG1 10 mg/mL concentrations were chosen for subsequent experiments, both individually and in combination, with sodium hyaluronate used as a comparator at the same concentration as non-animal HA (the selected concentration was 0.01%).

### 2.2. Absorption Evaluation of Non-Animal HA Alone and in Combination with Bifidobacterium longum in Intestinal In Vitro Model

**Figure 2:**
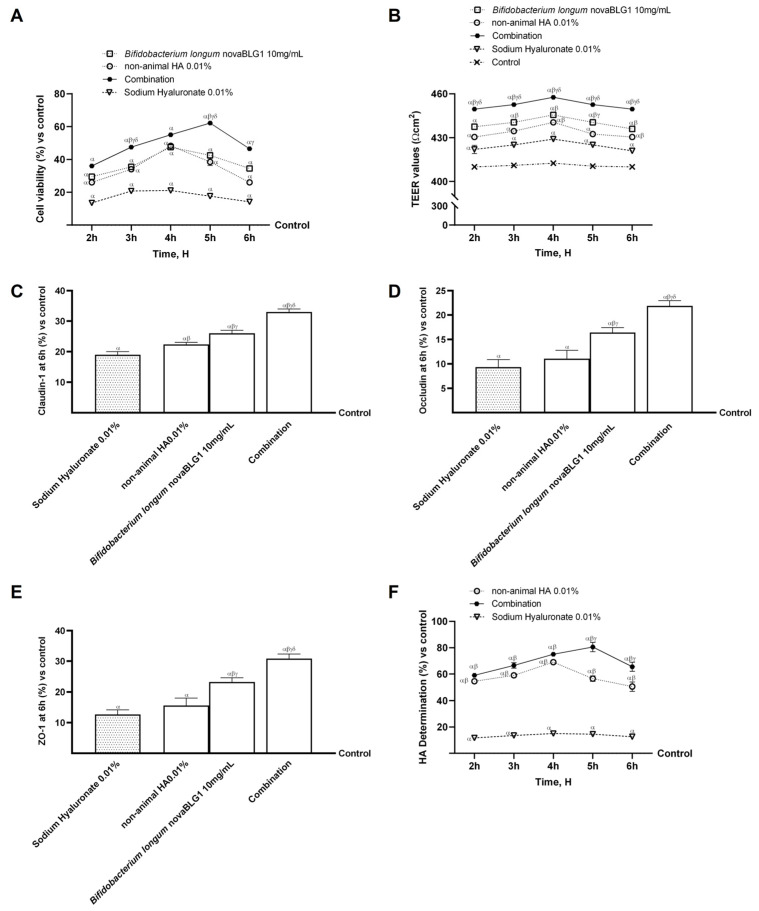
Effects of non-animal HA and probiotic strain on 3D intestinal barrier in vitro model. In (**A**), cell viability assessed by MTT is evaluated; in (**B**), TEER analysis is performed; in (**C**–**E**), the analysis of TJs measured by the Enzyme-Linked Immunosorbent Assay (ELISA) test (occludin, claudin-1, and ZO-1, respectively) at 6 h is depicted; and in (**F**), HA quantification performed by a specific ELISA kit is reported. Data are expressed as mean ± SD (%) of 5 independent experiments normalised to control (0% line). Combination = non-animal HA 0.01% + *Bifidobacterium longum* novaBLG1 10 mg/mL. α *p* < 0.05 vs. control; β *p* < 0.05 vs. sodium hyaluronate 0.01%; γ *p* < 0.05 vs. non-animal HA 0.01%; δ *p* < 0.05 vs. *Bifidobacterium longum* novaBLG1 10 mg/mL.

In an intestinal barrier in vitro model, additional studies were carried out to assess the effects of probiotics and non-animal HA, both separately and in combination, compared to sodium hyaluronate. To verify the safety of these molecules, cell viability, trans-epithelial electrical resistance (TEER) values, tight junction (TJ) levels, HA intestinal absorption, and apparent permeability coefficient (Papp) values were examined to demonstrate the ability of non-animal HA and *Bifidobacterium longum* novaBLG1 to work cooperatively. The results reported in Figure 2A show that cell viability was increased compared to the control (*p* < 0.05) after treatment with single agents and agents in combination. In particular, the combination exerted a more significant effect on intestinal cell viability at 5 h compared to single agents (about 61% vs. non-animal HA 0.01% and 46% vs. *Bifidobacterium longum* novaBLG1 10 mg/mL, *p* < 0.05) and sodium hyaluronate 0.01% (about 72%, *p* < 0.05). Subsequently, additional analyses were performed to evaluate intestinal barrier integrity by measuring TEER values. As reported in Figure 2B, the TEER evaluation shows that all tested substances maintained correct intestinal homeostasis (*p* < 0.05). Specifically, integrity analyses show that non-animal HA 0.01% and *Bifidobacterium longum* novaBLG1 10 mg/mL alone were able to maintain epithelial integrity by increasing the ion flux of paracellular exchanges through the intestinal epithelium better than sodium hyaluronate 0.01% (*p* < 0.05). Notably, the combination of non-animal HA 0.01% and *Bifidobacterium longum* novaBLG1 10 mg/mL enhanced the efficacy of the individual agents (*p* < 0.05). These data were confirmed with the TJ analysis (Figure 2C–E); non-animal HA 0.01% and *Bifidobacterium longum* novaBLG1 10 mg/mL alone exerted the most significant effects compared to sodium hyaluronate 0.01% for all the TJs tested (*p* < 0.05). Furthermore, the combination exerted the highest effect compared to single agents and sodium hyaluronate 0.01% regarding claudin-1 (about 47% vs. non-animal HA 0.01%, 30% vs. *Bifidobacterium longum* novaBLG1 10 mg/mL, and 74% vs. sodium hyaluronate 0.01%; *p* < 0.05), occludin (about 49% vs. non-animal HA 0.01%, 25% vs. *Bifidobacterium longum* novaBLG1 10 mg/mL, and 57% vs. sodium hyaluronate 0.01%; *p* < 0.05), and Zonula occludens-1 (ZO-1, about 49% vs. non-animal HA 0.01%, 33% vs. *Bifidobacterium longum* novaBLG1 10 mg/mL, and 59% vs. sodium hyaluronate 0.01%; *p* < 0.05) compared to the control value (reported as the 0 line, *p* < 0.05). Supplementary experiments were conducted to assess the permeability rate, evaluate the flow of non-electrolyte tracers (quantified as permeability coefficients), and determine the amount of non-animal HA that crossed the intestinal barrier to arrive at the specific target. Data obtained using the basolateral environment analysis, reported in Figure 2F and Table 1, support previous results since non-animal HA had higher values than sodium hyaluronate 0.01% with a maximum effect at 4 h (about 78%, *p* < 0.05). The combination showed a peak basolateral concentration of non-animal HA after 5 h of treatment, showing a significant percentage increase of about 82% compared to sodium hyaluronate 0.01% and about 30% compared to non-animal HA 0.01% (*p* < 0.05), suggesting modulatory activity in the absorption of non-animal HA by the probiotic strain.

### 2.3. Effects of Non-Animal HA and Bifidobacterium longum on Gut–Skin Axis

**Figure 3:**
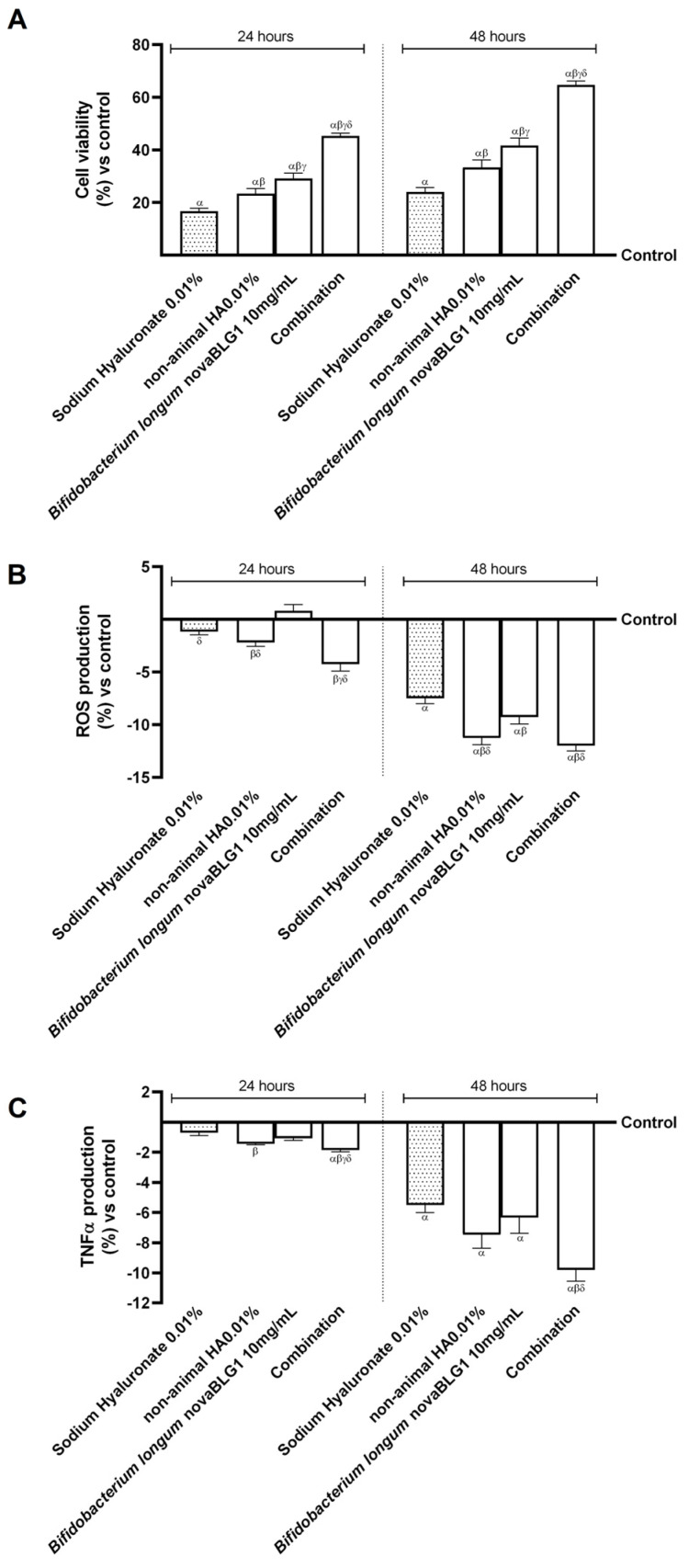
Effects of single agents and combination on gut–skin axis at 24 h and 48 h of treatment. (**A**) Cell viability assessed by MTT test; (**B**) ROS production analysis by cytochrome C reduction; (**C**) TNFα production analysis by ELISA kit. Data are expressed as mean ± SD (%) of 5 independent experiments normalised to control (0% line). Combination = non-animal HA 0.01% + *Bifidobacterium longum* novaBLG1 10 mg/mL. α *p* < 0.05 vs. control; β *p* < 0.05 vs. sodium hyaluronate 0.01%; γ *p* < 0.05 vs. non-animal HA 0.01%; δ *p* < 0.05 vs. *Bifidobacterium longum* novaBLG1 10 mg/mL.

Based on the results obtained previously, additional experiments were carried out on the gut–skin axis to explore the biological effects of combinations of non-animal HA 0.01% and *Bifidobacterium longum* novaBLG1 10 mg/mL and the single agents on the skin epithelium after intestinal passage. All data were collected after 24 h and 48 h of treatment and compared to sodium hyaluronate 0.01%. As represented in Figure 3A, NHEK cell viability was increased after 24 h and 48 h of treatment with the single agents and their combination compared to the control (*p* < 0.05). In particular, non-animal HA 0.01% and *Bifidobacterium longum* novaBLG1 10 mg/mL increased cell viability better than sodium hyaluronate 0.01% (about 40% and 75%, respectively, *p* < 0.05). The combination enhanced cell viability more than single agents and sodium hyaluronate (about 48% vs. non-animal HA 0.01%, 35.5% vs. *Bifidobacterium longum* novaBLG1 10 mg/mL, and 63% vs. sodium hyaluronate 0.01%; *p* < 0.05). The treatment at 24 h and 48 h exhibited a similar trend, with a more pronounced impact noticeable after 48 h compared to 24 h. This evidence was confirmed by a reactive oxygen species (ROS) production analysis at 24 h and 48 h of treatment, reported in Figure 3B. As was seen for cell viability, the combination in the study was able to reduce the production of ROS more effectively than single agents (about 6.5% vs. non-animal HA 0.01%, 29.5% vs. *Bifidobacterium longum* novaBLG1 10 mg/mL, *p* < 0.05) and sodium hyaluronate 0.01% (about 60%, *p* < 0.05). The most significant effect was observed at 48 h of treatment, confirming what was observed with for vitality. In addition, the inflammatory cytokine tumour necrosis factor alpha (TNFα) was analysed at 24 h and 48 h of treatment (Figure 3C). Once more, the combination examined demonstrated the capacity to suppress the inflammatory reaction by decreasing TNFα production with higher efficiency in comparison to individual agents (about 31% vs. non-animal HA 0.01%, 55% vs. *Bifidobacterium longum* novaBLG1 10 mg/mL, *p* < 0.05) and sodium hyaluronate 0.01% (about 78%, *p* < 0.05), especially 48 h post the initiation of treatment.

**Figure 4:**
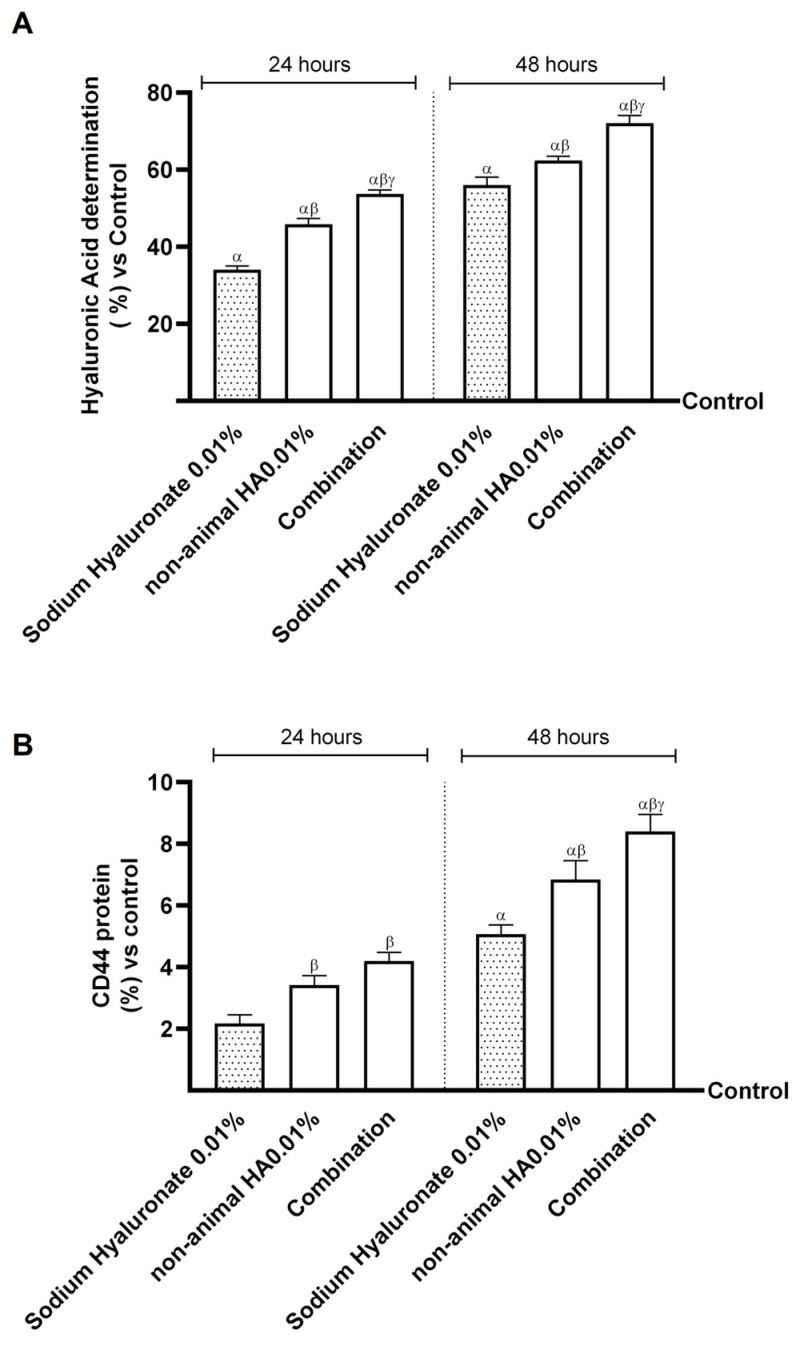
Effects of single agents and combination on gut–skin axis at 24 h and 48 h of treatment. In (**A**), HA determination was conducted by the ELISA kit; in (**B**), CD44 protein levels were analysed by the ELISA kit. Data are expressed as mean ± SD (%) of 5 independent experiments normalised to control (0% line). Combination = non-animal HA 0.01% + *Bifidobacterium longum* novaBLG1 10 mg/mL. α *p* < 0.05 vs. control; β *p* < 0.05 vs. sodium hyaluronate 0.01%; γ *p* < 0.05 vs. non-animal HA 0.01%.

Finally, to verify whether the probiotic strain could modulate intestinal non-animal HA absorption and the amount reached in the final target, two further experiments were performed 24 h and 48 h after treatment. The aim was to quantify the amount of non-animal HA and to determine the level of cluster of differentiation 44 (CD44) in the skin. As reported in Figure 4A, the amount of HA was very high after treatment with the combination compared to non-animal HA 0.01% and sodium hyaluronate 0.01% (about 15.5% vs. non-animal HA 0.01% and 28.5% vs. sodium hyaluronate 0.01%), in particular after treatment for 48 h. In addition, the data on CD44 levels after treatment for 24 h and 48 h with the study combination, shown in Figure 4B, show that the probiotic does not change the affinity of non-animal HA with the HA receptor but instead seems to improve it. Indeed, after treatment, the combination increased CD44 levels by about 23% compared to non-animal HA 0.01% and by about 66% compared to sodium hyaluronate 0.01%; also, in this case, the most significant effect was obtained at 48 h of treatment, confirming what was initially hypothesised about a possible intake every 48 h.

### 2.4. Effects of Non-Animal HA and Bifidobacterium longum novaBLG1 on Gut–Skin Axis After 144 h of Treatment

**Figure 5:**
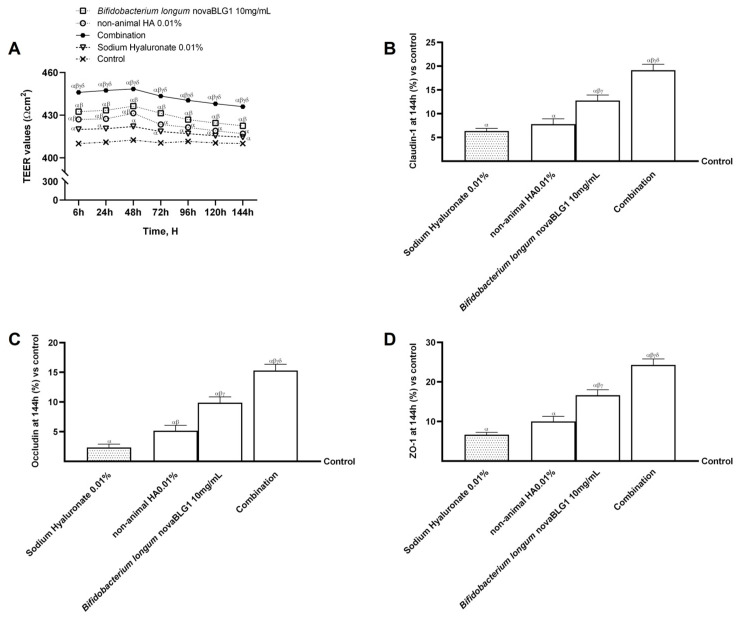
Effects of non-animal HA and probiotic strain on gut–skin axis after 144 h of treatment. In (**A**), TEER analysis is performed; in (**B**–**D**), the analysis of TJs measured by ELISA kit (occludin, claudin-1, and ZO-1, respectively) at 6 h is reported. Data are expressed as mean ± SD (%) of 5 independent experiments normalised to control (0% line). Combination = non-animal HA 0.01% + *Bifidobacterium longum* novaBLG1 10 mg/mL. α *p* < 0.05 vs. control; β *p* < 0.05 vs. sodium hyaluronate 0.01%; γ *p* < 0.05 vs. non-animal HA 0.01%; δ *p* < 0.05 vs. *Bifidobacterium longum* novaBLG1 10 mg/mL.

To evaluate the hypothesis that non-animal HA 0.01% combined with *Bifidobacterium longum* novaBLG1 10 mg/mL can significantly promote skin health over a treatment duration of 144 h, assessments of intestinal TEER values were carried out throughout the entire treatment phase, which spanned from 6 h to 144 h. These assessments were performed daily across the treatment timeline; considering that previous results indicated a peak agent response after 48 h, the protocol used consisted of a new administration every 48 h. In addition, TJ levels were analysed at 144 h to confirm the absence of irritability and the presence of intestinal integrity. As reported in Figure 5A, the TEER assessment indicates that all substances examined promoted optimal absorption while preserving the correct intestinal balance (*p* < 0.05). More specifically, passage through the intestinal epithelium reveals that non-animal HA 0.01% and *Bifidobacterium longum* novaBLG1 10 mg/mL alone maintained the integrity of the epithelium, enhancing the ionic flux of the paracellular exchanges through the intestinal epithelium more effectively than sodium hyaluronate 0.01% (*p* < 0.05). The combination exerted a significant effect compared to single agents and sodium hyaluronate 0.01% (*p* < 0.05). These results were validated through the TJ analysis. As illustrated in Figure 5B–D, non-animal HA 0.01% and *Bifidobacterium longum* novaBLG1 10 mg/mL alone exhibited the most notable impacts in comparison to sodium hyaluronate 0.01% across all the TJs examined (*p* < 0.05). Moreover, the combination showed the highest efficacy when compared to individual agents and sodium hyaluronate 0.01% concerning claudin-1 (approximately 59% vs. non-animal HA 0.01%, 33% vs. *Bifidobacterium longum* novaBLG1 10 mg/mL, and 67% vs. sodium hyaluronate 0.01%; *p* < 0.05), occludin (about 66% vs. non-animal HA 0.01%, 35% vs. *Bifidobacterium longum* novaBLG1 10 mg/mL, and 85% vs. sodium hyaluronate 0.01%; *p* < 0.05), and ZO-1 (approximately 59% vs. non-animal HA 0.01%, 31.5% vs. *Bifidobacterium longum* novaBLG1 10 mg/mL, and 72.5% vs. sodium hyaluronate 0.01%; *p* < 0.05) measured at 144 h. These data support the hypothesis that non-animal HA 0.01%, the probiotic, and their combination do not cause harmful effects even with prolonged treatment.

**Figure 6:**
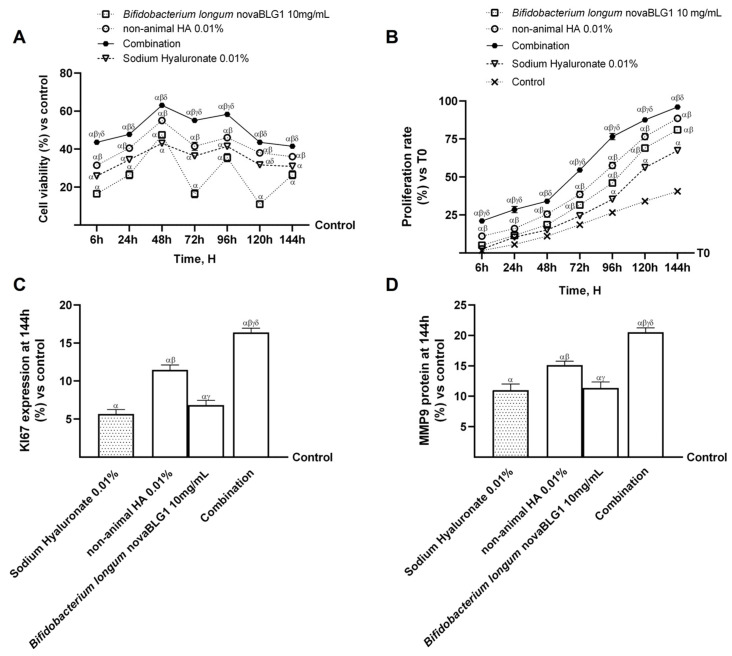
Effects of non-animal HA and probiotic strain on gut–skin axis. In (**A**), cell viability assessed by MTT test is evaluated from 6 h to 144 h; in (**B**), cell proliferation by crystal violet staining is evaluated from 6 h to 144 h; in (**C**), Ki67 expression is measured by ELISA kit at 144 h; in (**D**), MMP9 protein levels are measured by ELISA kit at 144 h. Data are expressed as mean ± SD (%) of 5 independent experiments normalised to control (0% line). Combination = non-animal HA 0.01% + *Bifidobacterium longum* novaBLG1 10 mg/mL. α *p* < 0.05 vs. control; β *p* < 0.05 vs. sodium hyaluronate 0.01%; γ *p* < 0.05 vs. non-animal HA 0.01%; δ *p* < 0.05 vs. *Bifidobacterium longum* novaBLG1 10 mg/mL.

Based on the results obtained previously, further experiments were carried out on the gut–skin axis to study the biological effects of the combination of non-animal HA 0.01% and *Bifidobacterium longum* novaBLG1 10 mg/mL and the individual agents on the skin epithelium followed by intestinal passage after a treatment period of 6 h to 144 h, comparing the effects with those of sodium hyaluronate 0.01%. As shown in Figure 6A, cell viability was increased over the 144 h treatment period with the single agents and the combination compared to the control and sodium hyaluronate 0.01% (*p* < 0.05). In particular, non-animal HA 0.01% and *Bifidobacterium longum* novaBLG1 10 mg/mL alone were able to increase cell viability better than sodium hyaluronate 0.01% (*p* < 0.05); also, the combination enhanced cell viability more than single agents and sodium hyaluronate 0.01% (*p* < 0.05). An analysis of cell proliferation confirmed this finding and was carried out throughout treatment between 6 h and 144 h, as shown in Figure 6B. The combination was able to increase cell proliferation more effectively than the single agents (about 16% vs. non-animal HA 0.01%, 8% vs. *Bifidobacterium longum* novaBLG1 10 mg/mL, *p* < 0.05) and sodium hyaluronate 0.01% (about 29.7%, *p* < 0.05) after 144 h of treatment. Further experiments were performed at 144 h of treatment to analyse the levels of MMP9, an important member of the group of metalloproteases that form the extracellular matrix, [32] and antigen Kiel 67 (Ki67) expression, a cellular marker for studying keratinocyte proliferation [33], respectively. As reported in Figure 6C,D, the data showed that all substances tested induced significant changes in Ki67 expression and MMP9 levels compared to the control (*p* < 0.05). Ki67 is a cellular marker for studying keratinocyte proliferation [34], and the analysis showed that both non-animal HA 0.01% and *Bifidobacterium longum* novaBLG1 10 mg/mL induced a significant increase in Ki67 compared to the control (*p* < 0.05); as expected, the combination of these two agents amplified these effects (about 30% vs. non-animal HA 0.01%, 58.5% vs. *Bifidobacterium longum* novaBLG1 10 mg/mL, and 65% vs. sodium hyaluronate 0.01%; *p* < 0.05). The analysis of MMP9 was also evaluated, indicating that the substances used increased MMP9 levels. This effect was more evident when non-animal HA 0.01% and *Bifidobacterium longum* novaBLG1 10 mg/mL were used in combination (about 26% vs. non-animal HA 0.01%, 45% vs. *Bifidobacterium longum* novaBLG1 10 mg/mL, and 46% vs. sodium hyaluronate 0.01%; *p* < 0.05).

**Figure 7:**
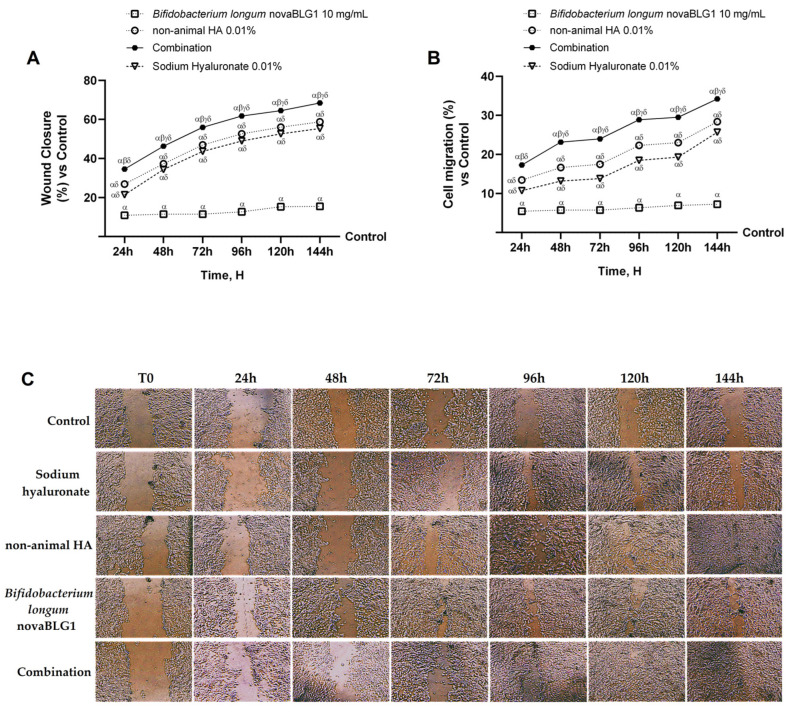
Effects of non-animal HA and probiotic strain on gut–skin axis. (**A**) Wound closure analysis by wound healing test from 24 h to 144 h; (**B**) cell migration evaluation by cell migration assay from 24 h to 144 h; (**C**) representative pictures of wound healing with each treatment at T0 and after 24 h, 48 h, 72 h, 96 h, 120 h, and 144 h taken through microscopy at original magnification of ×20. Data are expressed as mean ± SD (%) of 5 independent experiments normalised to control (0% line). Combination = non-animal HA 0.01% + *Bifidobacterium longum* novaBLG1 10 mg/mL. α *p* < 0.05 vs. control; β *p* < 0.05 vs. sodium hyaluronate 0.01%; γ *p* < 0.05 vs. non-animal HA 0.01%; δ *p* < 0.05 vs. *Bifidobacterium longum* novaBLG1 10 mg/mL.

A wound healing and cellular migration assessment was conducted to validate the data acquired from the proliferation assays (Figure 7). As depicted in Figure 7A, sodium hyaluronate 0.01%, non-animal HA 0.01%, *Bifidobacterium longum* novaBLG1 10 mg/mL, and the combination demonstrated significant enhancements in wound healing by 55.4%, 58.7%, 15.5%, and 68.5%, respectively, in comparison to the control following a treatment duration of 144 h. Notably, the combination treatment exhibited superior efficacy in promoting wound healing compared to the individual agents after the 144 h treatment period (19% vs. sodium hyaluronate 0.01%, 14% vs. non-animal HA 0.01%, and 77% vs. *Bifidobacterium longum* novaBLG1). Correspondingly, the results from the migration assay support the earlier conclusions drawn from the wound healing assay (Figure 7B). Once more, sodium hyaluronate 0.01%, non-animal HA 0.01%, *Bifidobacterium longum* novaBLG1 10 mg/mL, and the combination demonstrated accelerated wound healing efficacy of 25.7%, 28.3%, 7.25%, and 34.2%, respectively, in comparison to the control group after a treatment duration of 144 h. Notably, the combination exhibited superior efficacy in promoting wound healing compared to the individual agents following the 144 h treatment period (25% vs. sodium hyaluronate 0.01%, 17% vs. non-animal HA 0.01%, and 54% vs. *Bifidobacterium longum* novaBLG1 10 mg/mL).

**Figure 8:**
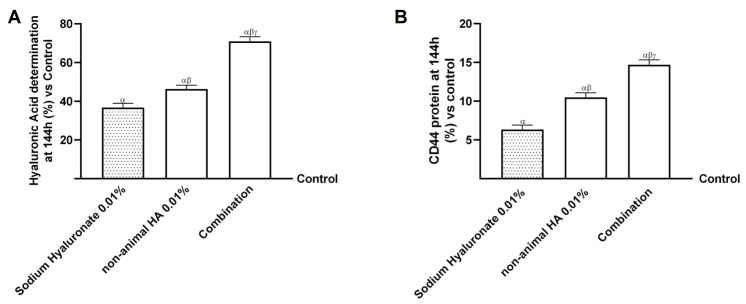
Effects of non-animal HA and probiotic strain on gut–skin axis after 144 h of treatment. In (**A**), HA determination is performed using the ELISA kit, and in (**B**), CD44 protein levels are analysed using the ELISA kit. Data are expressed as mean ± SD (%) of 5 independent experiments normalised to control (0% line). Combination = non-animal HA 0.01% + *Bifidobacterium longum* novaBLG1 10 mg/mL. α *p* < 0.05 vs. control; β *p* < 0.05 vs. sodium hyaluronate 0.01%; γ *p* < 0.05 vs. non-animal HA 0.01%.

In conclusion, to verify whether *Bifidobacterium longum* novaBLG1 could modulate intestinal non-animal HA absorption and the amount reached in the final target, two further experiments were performed 144 h after treatment. The aim was to quantify the amount of HA after treatment with all agents and determine the specific CD44 receptor levels in the skin. As reported in Figure 8A, the amount of HA was very high after treatment with the combination compared to non-animal HA 0.01% alone and sodium hyaluronate 0.01% (about 35% vs. non-animal HA 0.01% and 48% vs. sodium hyaluronate 0.01%, respectively; *p* < 0.05). In addition, data on CD44 levels after 144 h of treatment with the combination show that the probiotic does not change the affinity of non-animal HA to its receptor but appears to improve it (Figure 8B). Indeed, after treatment, the combination increased CD44 levels by about 28.5% compared to non-animal HA 0.01% alone and by about 57% compared to sodium hyaluronate 0.01%.

**Figure 9:**
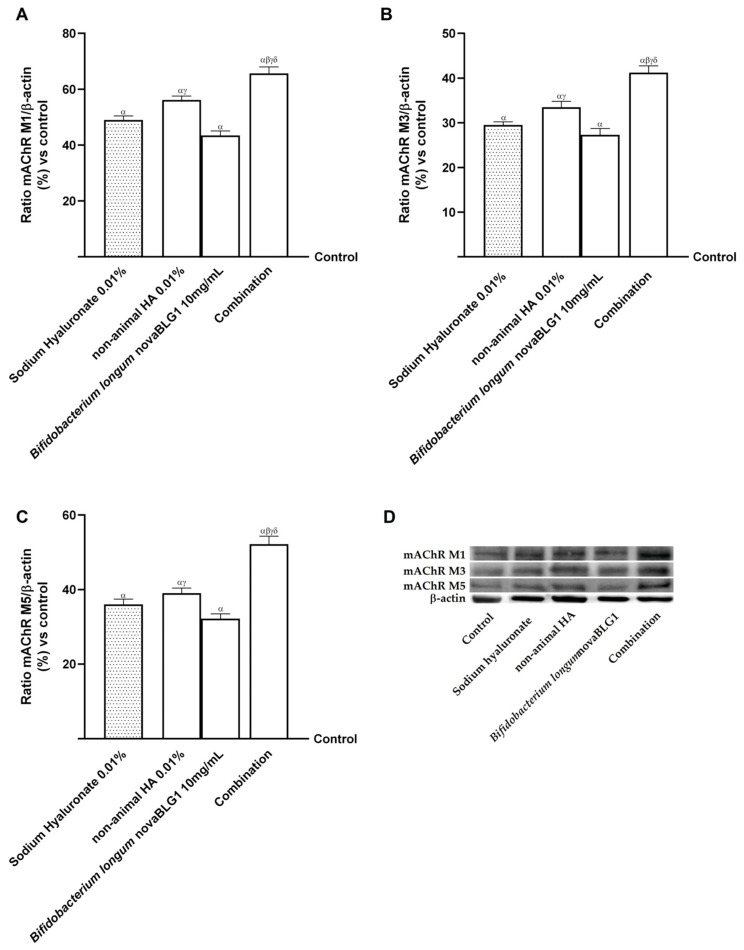
Effects of non-animal HA and probiotic strain on gut–skin axis after 144 h of treatment. (**A**) mAChR M1 expression; (**B**) mAChR M3 expression; (**C**) mAChR M5 expression obtained by Western blot analysis. In (**D**), an example of the densitometric analysis of the specific Western blot is reported. Data are expressed as mean ± SD (%) of 5 independent experiments normalised to control (0% line). Combination = non-animal HA 0.01% + *Bifidobacterium longum* novaBLG1 10 mg/mL. α *p* < 0.05 vs. control; β *p* < 0.05 vs. sodium hyaluronate 0.01%; γ *p* < 0.05 vs. non-animal HA 0.01%; δ *p* < 0.05 vs. *Bifidobacterium longum* novaBLG1 10 mg/mL.

Finally, since keratinocytes express various non-neuronal cholinergic receptor isoforms [35], muscarinic acetylcholine receptor type 1 (mAChR M1), muscarinic acetylcholine receptor type 3 (mAChR M3), and muscarinic acetylcholine receptor type 5 (mAChR M5) were also analysed. As reported in Figure 9, non-animal HA 0.01% increased all three isoforms’ expression (*p* < 0.05). Treatment with *Bifidobacterium longum* novaBLG1 10 mg/mL and non-animal HA 0.01% increased the involvement of mAChR M1, mAChR M3, and mAChR M5 in the healing mechanism activated; this improvement was amplified when *Bifidobacterium longum* novaBLG1 10 mg/mL and non-animal HA 0.01% were combined compared to the single agents and sodium hyaluronate 0.01% (*p* < 0.05).

## 3. Discussion

With the rising prevalence of an elderly population in numerous nations, the psychological and social repercussions of skin ageing intensify the need for strategies to address this issue [36]. In recent years, the demand for anti-ageing products has increased significantly, and the anti-ageing group tends to be younger, in addition to the continuous improvement of material life and the increase in the average lifespan [37]. Topical applications, energy-based treatments, chemical peels, injectable treatments, skin rejuvenation techniques, and surgical procedures are among clinics’ most frequently employed anti-ageing strategies [38]. These procedures increase skin firmness and encourage collagen production, which helps prevent ageing [38].

HA is a unique topical agent and filler, known for its natural composition, biocompatibility, reversibility, and adaptability, which can support anti-ageing strategies [37]. HA is a popular ingredient in cosmetics because it is able to retain a lot of water and is used in various forms, such as creams, gels, and fillers [39]. Studies show that HA supplements help the skin produce more collagen and water, reducing wrinkles and promoting skin renewal [36,39]. The use of nutraceuticals, like HA, is rising in the treatment of skin issues. Research has explored HA’s cosmetic effects, focusing on its impact on skin hydration and tissue growth [40]. Its molecular weight affects its biological activity and receptor binding, influencing cell functions [41,42].

There is growing evidence that the microbes in our gut can influence diseases outside of the gastrointestinal tract. The gastrointestinal tract is home to many microbes that affect our health, and the skin, with its glands and large surface area, can also interact with these microbes. Recent progress in the microbiome field is showing how we can change the gut or skin microbiome to improve skin health in the future [43]. Many probiotics have antioxidant, anti-ageing, and skin moisturising properties, as well as the capacity to improve gut epithelial defence.

The body can better absorb HA when it is combined with probiotics. So, using HA together with probiotics could be a great way to make the most of the health benefits of HA [44].

In this study, a 0.01% concentration of non-animal HA, a new vegan HA form, in combination with the probiotic strain *Bifidobacterium longum* novaBLG1 10 mg/mL (equivalent to 1 × 10^9^ cfu), was able to maintain cell viability without damaging or irritating the intestinal tissue. This confirms the hypothesis that oral supplementation of non-animal HA and probiotics can be used to maintain skin well-being. 

The results of a 3D model mimicking intestinal absorption demonstrate that the administration of non-animal HA 0.01% is feasible, as it is efficiently absorbed and biodistributed in the skin, where it exerts its biological function. Indeed, non-animal HA 0.01% reaches higher plasma levels than sodium hyaluronate 0.01% in the control group (*p* < 0.05), especially when combined with probiotics. This supports the hypothesis that this combination enhances absorption during the physiological period of intestinal digestion and improves bioavailability. Moreover, treatment with this combination revealed that a significant portion of non-animal HA 0.01% was absorbed without damaging the intestinal epithelium. This is important since HA plays a role in reducing permeability by improving tight junction proteins—claudins, occludin, and ZO-1 proteins—which are necessary for epithelial barrier activity [45]. These three proteins are pivotal because ZO-1 connects claudin and occludin to the cytoskeleton, indicating good gut barrier function [46]. Furthermore, it has been demonstrated that non-animal HA 0.01% can maintain epithelial integrity and ionic exchanges across the intestinal barrier, suggesting that this proteoglycan, combined with probiotics, can cross the cell monolayer without negatively altering the epithelium. These data have been shown to be in accordance with recent research that emphasises the significance of certain peptides (like HA) and probiotics in enhancing gut health, bolstering the immune system, and lowering the likelihood of illness. The personal care sector incorporates probiotics in creams, serums, and drinks, as the synergy between probiotics and peptides such as HA boosts the effectiveness of the components, leading to improved antioxidant capabilities, defence against environmental harm, and enhanced anti-ageing benefits [47].

This work’s second important purpose was to test non-animal HA 0.01%’s ability to stimulate keratinocyte biological activity under physiological conditions. As expected, non-animal HA 0.01% stimulated cell viability and reduced ROS and TNFα production without causing adverse effects compared to conventional sodium hyaluronate 0.01%. This effect was amplified after the addition of the probiotic strain. Furthermore, this combination could secure a higher percentage of non-animal HA 0.01% at the keratinocyte level than sodium hyaluronate 0.01% without altering its affinity for the CD44 receptor, which was present in high concentrations after treatment with the combination. The current findings indicate that markedly heightened expression of CD44 may be correlated with its role in the maintenance and preservation of the epidermal barrier in affected individuals [48].

Clinical studies in the last few years have demonstrated that oral HA treatment can significantly improve skin conditions [11]. Hsu et al., Sato et al. [49,50,51], and Yoshida et al. [52] performed randomised, double-blind, placebo-controlled experiments on individuals with dry skin. The findings demonstrated that oral administration of HA significantly reduced skin dryness and increased skin hydration. Kawada et al. [53] treated 61 patients with dry skin using HMW-HA and found a comparable improvement in skin hydration. Additional research by Schwartz et al. [54] revealed that oral HA treatment could reduce facial skin ageing symptoms. According to the findings, HA may be pivotal in skin well-being. For this reason, to validate the hypothesis regarding the potential administration of the compound for 144 h, the integrity experiments were reiterated over a period ranging from 6 h to 144 h to prevent any negative consequences associated with prolonged compound usage. The administration of the compound every alternate day for 144 h at the gastrointestinal level did not yield any adverse reactions, thereby ensuring the preservation of the cellular monolayer’s integrity, as evidenced by the values of TEER and the levels of TJ proteins. Subsequent investigations focused on assessing the capability of non-animal HA 0.01% combined with the probiotic to enhance the viability and proliferation of keratinocyte cells while also increasing the expression of Ki67, the levels of MMP9, the concentration of HA, and the levels of CD44. The compound demonstrated remarkable and positive effects that exceeded those observed with sodium hyaluronate 0.01% in all parameters studied. This confirms the action of the combination at the level of the skin without any loss of effect after intestinal transit, leading to the hypothesis that these results can also be obtained in vivo. Finally, an examination was conducted of the levels of muscarinic receptors M1, M3, and M5, as they play a role in stimulating MAP kinase proteins in keratinocytes [35]. In our findings, NHEK cells demonstrate the involvement of receptors in the skin repair mechanism (*p* < 0.05 vs. control) in terms of proliferation, especially after combination treatment. Taken together, these results suggest that combining non-animal HA 0.01% and *Bifidobacterium longum* novaBLG1 10 mg/mL (corresponding to daily consumption of 1 × 10^9^ cfu) is the best choice for promoting skin well-being, supposing its oral administration.

## 4. Materials and Methods

### 4.1. Agent Preparation

White Tremella (silver ear) is a traditional Chinese medicinal mushroom that is a source of non-animal HA [55]. The extraction and production method produces an innovative HA, patented by Vivatis Pharma GBHE (Grüner Deich 1–3, 20097 Hamburg, Germany) (Patent No. WO2021/250566). The procedure includes sieving, crushing, and alcohol solution-based extraction, purification, and refining. Next, the powder is packaged, examined and stored [56,57]. To confirm the mechanism of action of non-animal HA, sodium hyaluronate (Merck Life Science, Rome, Italy), which is commonly available commercially, was also tested as a reference.

All of these substances were prepared directly in Dulbecco’s Modified Eagle’s Medium (DMEM, Merck Life Science, Rome, Italy) without phenol red and supplemented with 0.5% foetal bovine serum (FBS, Merck Life Science, Rome, Italy), 2 mM L-glutamine (Merck Life Science, Rome, Italy) and 1% penicillin–streptomycin (Merck Life Science, Rome, Italy) for biological analysis, both at a range of concentrations.

In addition, non-animal HA was combined with a probiotic strain selected after efficacy screening. The probiotic strain selected was *Bifidobacterium longum* novaBLG1 (DSM 34338, donated by Probionova SA, Lugano, Switzerland) dissolved in phosphate-buffered saline (PBS) 1× and tested in a concentration range of 10 µg/mL to 10mg/mL (equivalent to a probiotic daily consumption of 1 × 10^6^–1 × 10^9^ cfu).

### 4.2. Cell Culture

Human intestinal epithelial cells, Caco-2, purchased from the American Type Culture Collection (ATCC, Manassas, VA, USA), were cultured in Advanced Dulbecco’s Modified Eagle’s Medium/Nutrient F-12 Ham’s (Adv DMEM-F12; GIBCO^®^ ThermoFisher Scientific, Waltham, MA, USA) containing 10% FBS, 2 mM L-glutamine, and 1% penicillin–streptomycin and maintained in a 37 °C incubator at 5% CO_2_ [58]. The cells employed in the experiments were at 26 to 32 passages to maintain the integrative paracellular permeability and transport characteristics [59] to uphold similarity with intestinal absorption after oral ingestion in the human body. The cells were plated differently to perform different experiments, including 1 × 10^4^ cells in 96-well plates to study cell viability by a 3-(4,5-Dimethylthiazol-2-yl)-2,5-diphenyltetrazolium bromide (MTT)-based In Vitro Toxicology Assay Kit (Merck Life Science, Rome, Italy), and 2 × 10^4^ cells on a 6.5 mm Transwell^®^ with a 0.4 μm pore polycarbonate membrane insert (Corning Costar, New York, NY, USA) in a 24-well plate to perform an absorption and integrity study by analysing TEER values and TJ protein levels [60]. Especially for the creation of a gut–skin axis, at the end of the maturation period, Transwell^®^ inserts seeded with intestinal cells were placed in connection with a monolayer of normal human epidermal keratinocytes. Before the cell viability study, cells were incubated for 8 h with DMEM without red phenol and FBS (GIBCO^®^, ThermoFisher Scientific, Waltham, MA, USA) but containing 1% penicillin–streptomycin, 2 mM L-glutamine, and 1 mM sodium pyruvate (Merck Life Science, Rome, Italy) to synchronise them. On the other hand, before Transwell^®^ stimulation, on the apical side, the medium was brought to pH 6.5 as the pH in the lumen of the small intestine, while pH 7.4 on the basolateral side represented blood [60].

Normal human epidermal keratinocytes (NHEKs) derived from neonatal foreskin were acquired from Lonza (Basel, Switzerland) and cultured in a keratinocyte basal medium (KBM medium, Lonza, Basel, Switzerland) supplemented with keratinocyte growth supplements (KGM2, Lonza, Basel, Switzerland) comprising insulin, human epidermal growth factor, bovine pituitary extract, hydrocortisone, epinephrine, transferrin, and gentamicin/amphotericin B. The cells were maintained in a controlled environment at 37 °C and 5% CO_2_ until they achieved 70–80% confluence. The experimental procedures were carried out using cells in the proliferation phase at passage two or three, aiming to replicate in vitro the conditions observed in human skin [61]. This cell line is widely utilised in the literature as an in vitro model to research cell proliferation, migration, and differentiation [62]. For the experiments, 1 × 10^3^ cells were plated on a 96-well plate to study cell viability by the MTT test and crystal violet staining, and gut–skin interaction was evaluated in a 24-well plate. Specifically, keratinocytes were seeded at a density of 5 × 10^5^ cells per well 2 days before the intestinal epithelium reached maturation. When the cells were matured, the intestinal barrier and keratinocytes were placed in contact to quantify the concentration of hyaluronic acid crossing the intestinal barrier and to analyse the levels of the specific hyaluronic acid receptor (CD44) in keratinocytes. In addition, the ELISA kit was used and Western blot analysis was performed on the gut–skin axis to explore intracellular pathways such as matrix metalloproteinase-9 (MMP9), antigen Ki67, mAChR M1, mAChR M3, and mAChR M5. NHEK cells were placed for 24 h in a keratinocyte KBM medium without hydrocortisone or transferrin before treatments to synchronise them.

### 4.3. Experimental Protocol

The experiments were divided into three phases to assess whether the oral administration of non-animal HA alone or in conjunction with a probiotic strain could be a beneficial treatment for skin health.

In the first phase, Caco-2 cells were used to perform a dose–response study of different concentrations of non-animal HA (range: 0.001% to 0.1%) [60] and the probiotic strain *Bifidobacterium longum* novaBLG1 (10 µg/mL to 10 mg/mL, equivalent to 1 × 10^6^–1 × 10^9^ cfu) to select the best concentration to be used in subsequent experiments. Then, an in vitro intestinal model was recreated to assess non-animal HA’s ability to cross the intestinal barrier, excluding cytotoxicity. In more detail, non-animal HA was tested alone and in combination with a probiotic strain, with the intention of assessing whether the combination could have different absorption kinetics due to modulation by the probiotic. For this reason, viability (by the MTT assay) and ROS production (through the reduction of cytochrome C) analyses were carried out following treatment with non-animal HA alone and in combination with the probiotic strain, and the effects were compared with sodium hyaluronate used at the same dosage as non-animal HA. Subsequently, in the same in vitro model, non-animal HA and probiotics were tested, alone and in combination, in comparison with sodium hyaluronate to verify intestinal integrity through TEER measurement, TJ analysis (claudin-1, occludin and ZO-1) by the ELISA kit, and a permeability assay by Papp measurement, also analysing the total amount of non-animal HA that had crossed the intestinal barrier. For all these experiments, cells were treated in a time-dependent manner from 2 to 6 h, as reported in the literature [58].

In the second phase, the characteristics of non-animal HA alone and in combination with the probiotic were examined to determine their direct impact on keratinocytes by assessing various parameters and elucidating the underlying mechanism. To achieve this, a gut–skin axis was created using the Transwell^®^ system (Corning Costar, New York, NY, USA): intestinal cells were plated on the Transwell^®^ membrane until maturation and monolayer formation, while keratinocytes were cultured on the bottom of the plate’s wells. This system enables communication between the gut and the skin, reproducing what could happen in the body. Thus, each stimulation was administered on the apical side and metabolised by the gut cells and then on NHEK cells to evaluate cell viability (by MTT assay), ROS production (through the reduction of cytochrome C), inflammatory cytokine levels (TNFα), and CD44 concentrations, and to measure HA at the basal level after 24 and 48 h.

Ultimately, in the third phase, to evaluate the potential for extended oral intake over time, all treatments in the study were assessed for up to 144 h using an alternate-day administration protocol, informed by findings from phase two. In particular, the gut–skin axis was re-established, as explained before, to track TEER values over a duration ranging from 6 h to 144 h and to examine the levels of TJ at the 144 h treatment mark. This was conducted to avert the possibility that prolonged administration could lead to intestinal irritability. In this phase, analyses were also conducted on keratinocytes; specifically, analyses were conducted of cell viability (by MTT assay), cell proliferation (by crystal violet staining), wound healing, and cell migration over time from 6 h to 144 h. Lastly, the mechanisms contributing to the maintenance of well-being and skin homeostasis, such as Ki67, MMP9, mAChR M1, mAChR M3, and mAChR M5, were investigated after 144 h of treatment (stimulation every alternate day), along with the levels of CD44 and HA captured by keratinocytes.

### 4.4. In Vitro Intestinal Barrier Model

Following a standard procedure reported in the literature [62] and accepted by the European Medicines Agency (EMA) and Food and Drug Administration (FDA) [63,64], an in vitro intestinal barrier model was developed using the Transwell^®^ system to estimate the absorption, metabolism, and bioavailability of several substances after oral intake in humans. This investigation aimed to assess the potential of non-animal HA in traversing the intestinal barrier to reach the designated target location.

The experimental procedures began with seeding Caco-2 cells, following established protocols, and culturing them in a complete growth medium for 21 days. Throughout this period, the culture medium was systematically exchanged between the basolateral and apical compartments to sustain optimal growth conditions and promote proper cellular differentiation. This systematic approach ensured the integrity of the cell monolayer, effectively mimicking the physiological environment relevant to subsequent experimental protocols [65]. On the 21st day, when TEER values were ≥400 Ω∙cm^2^ [58], absorption analysis was started. Before stimulation, the medium was adjusted to pH 6.5, the pH in the lumen of the small intestine, and pH 7.4 on the basolateral side represented blood [59]. To ensure the levels had stabilised, the cells were kept at 37 °C with 5% CO_2_ for 15 min before the experiment began. The cells were stimulated with all agents for 2 to 6 h before the analyses, including the Papp analysis, which was conducted with the following formula [60]:

Papp = dQ/dt ⇥ 1/m0 ⇥ 1/A ⇥ V Donor

dQ: amount of substance transported (nmol or μg);

dt: incubation time (s);

m0: amount of substrate applied to donor compartment (nmol or μg);

A: surface area of Transwell^®^ membrane (cm^2^);

V Donor: volume of donor compartment (cm^3^).

Negative controls without cells were tested to exclude Transwell^®^ membrane influence.

### 4.5. In Vitro Toxicology Assay Kit

Cell viability was confirmed after each stimulation for both cell types using the In Vitro Toxicology Assay Kit (Merck Life Science, Rome, Italy), according to a conventional technique documented in the literature [66]. A spectrometer (Infinite 200 Pro MPlex, Tecan, Männedorf, Switzerland) was used to measure the absorbance of all solubilised samples (treated and untreated) at 570 nm with correction at 690 nm. The data were compared to the control sample. Results are presented as the mean ± SD (%) of viable cells compared to the control (untreated samples) of five independent experiments performed in triplicate.

### 4.6. Human Claudin-1 ELISA Kit

Human claudin-1 was quantified in Caco-2 lysates using an ELISA kit (Cusabio Technology LLC, Huston, TX, USA), according to the manufacturer’s instructions [62]. The cells were lysed with cold PBS (Merck Life Science, Rome, Italy) 1× and centrifuged at 1500× *g* for 10 min at 4 °C. The ELISA plate was loaded with 100 μL of each sample and incubated at 37 °C for 2 h. The plate was then cleaned, and 100 μL of Biotin-antibody was added to the wells and incubated for 1 h at 37 °C. After the wells had been cleaned, the samples were incubated for 1 h at 37 °C with 100 L of HRP-avidin added to each well. After the samples were mixed with 90 µL of TMB Substrate, the plate was incubated at 37 °C for 20 min without light. After the reaction was halted with 50 μL of Stop Solution, the plate was analysed at 450 nm using a spectrophotometer (Infinite 200 Pro MPlex, Tecan, Männedorf, Switzerland). The results are displayed as a percentage (mean ± SD) compared to the control (0 line) of five independent experiments performed in triplicate, and the concentration is expressed as pg/mL when data are compared to the standard curve (range 0 to 1000 pg/mL).

### 4.7. Human Occludin ELISA Kit

The Human Occludin ELISA kit (OCLN kit, MyBiosource, San Diego, CA, USA) analysed the presence of occludin in Caco-2 cell lysates, according to the manufacturer’s instructions [67]. Caco-2 cells were lysed with PBS 1×, and then transferred to a 100 µL strip well and incubated for 90 min at 37 °C. Strips were treated with 100 µL of Detection Solution A for 45 min at 37 °C, and then washed with Wash Solution and incubated with 100 µL of Detection Solution B for another 45 min. After the incubation, 90 μL of Substrate Solution was added and incubated for 20 min at 37 °C in the dark. Finally, 50 μL of Stop Solution was added to stop the enzymatic process, and the plate was analysed by a spectrophotometer (Infinite 200 Pro MPlex, Tecan, Männedorf, Switzerland) at 450 nm. The concentration is provided as pg/mL in comparison to a standard curve (from 0 to 1500 pg/mL), and the findings are expressed as a percentage (mean ± SD) versus the control (0 line) of five independent experiments performed in triplicate.

### 4.8. Human Tight Junction Protein 1 ELISA Kit

The Human Tight Junction Protein 1 (ZO-1) ELISA kit (MyBiosource, San Diego, CA, USA) was used in Caco-2, following the manufacturer’s instructions [62]. The cells underwent two cycles of freezing and thawing. They were washed briefly with ice-cold PBS 1× (Merck Life Science, Rome, Italy). The cell lysates were centrifuged for five minutes at 5000 g at 4 °C. Then, 100 µL of each sample was collected and incubated on an ELISA plate for 90 min at 37 °C. After washing, 100 µL of Detection Solution A was applied to each well, and the plate was incubated for 45 min at 37 °C. A total of 100 µL of Detection Solution B was added to the samples after the wells had been cleaned and incubated for 45 min at 37 °C in the dark. At the end of the incubation period, the wells were once more cleaned, 90 µL of Substrate Solution was added to each well, and the plate was incubated for 20 min at 37 °C in the dark. After 50 µL of Stop Solution was added, a spectrophotometer read the plate (Infinite 200 Pro MPlex, Tecan, Männedorf, Switzerland) at 450 nm. The data are represented as a percentage (mean ± SD) compared to the control (0 line) of five independent experiments performed in triplicate, and the concentration is expressed as pg/mL when comparing the data to the standard curve (0–1000 pg/mL).

### 4.9. Gut–Skin Axis

At two days before the completion of the gut cell maturation process, 5 × 10^5^ keratinocytes were seeded into wells of a 24-well plate and maintained at 37 °C until a cell monolayer was formed. Following a two-day maturation period for both the gut and keratinocytes, the Transwell^®^ insert containing the gut cells was placed within the 24-well plate seeded with the keratinocytes. This enabled the gut to be connected to the skin tissue, thus completing the gut–skin axis model for the treatment under study [68].

### 4.10. ROS Production

A traditional approach based on cytochrome C reduction was employed to determine the rate of superoxide anion release in NHEK cells [60]. Both treated and untreated cells received 100 μL of cytochrome C and 100 μL of superoxide dismutase (all chemicals were given by Merck Life Science, Rome, Italy) for 30 min in an incubator. A spectrometer (Infinite 200 Pro MPlex, Tecan, Männedorf, Switzerland) was used to detect the absorbance in culture supernatants at 550 nm, and O_2_ is expressed as the mean ± SD (%) of nanomoles of reduced cytochrome C per microgram of protein compared to the control (0 line) [45] of five independent experiments performed in triplicate.

### 4.11. TNFα Production ELISA Kit

TNFα quantification in NHEK cell supernatants was conducted using the TNFα ELISA kit (Merck Life Science, Rome, Italy) according to the manufacturer’s instructions [69]. Briefly, 100 μL of each sample (NHEK supernatants) was added to each well of a 96-well ELISA plate and incubated for 2 h at room temperature. After incubation, the wells were washed five times with a wash buffer, after which 100 μL of biotinylated anti-TNFα was added to each well and incubated for 2 h at room temperature. After the time had elapsed, the solution in each well was aspirated, and the wells were washed 5 times. Then, 100 μL of streptavidin-HRP was added to each well and incubated at room temperature for 1 h. After washing, 100 μL of chromogen solution was added to each well and incubated at room temperature and in the dark for 30 min. The absorbance of samples was measured at 450 nm using a plate reader (Infinite 200 ProMPlex, Tecan, Männedorf, Switzerland). The results were derived using a calibration curve (range: 24.58 pg/mL–6000 pg/mL) and are expressed as mean values ± SD (%) relative to the control (0 line) of five independent experiments performed in triplicate.

### 4.12. Hyaluronic Acid ELISA Kit

Both cell types (Caco-2 and NHEK) were lysed once with 100 μL of cold PBS 1× at the end of stimulation, and total HA was quantified according to the instructions of the Hyaluronic Acid ELISA kit (CloudClone, Houston, TX, USA). Briefly, 50 μL of the sample and reagent A were added to each well, and the plate was gently shaken and then incubated at 37 °C for 1 h. After the wells were washed three times, 100 μL of reagent B was added, and the plate was incubated at 37 °C for 30 min. Then, 90 μL of Substrate Solution was added, and the plate was incubated for 20 min at 37 °C. Finally, 50 μL of Stop Solution was added immediately before measuring the samples at 450 nm in a spectrophotometer (Infinite 200 Pro MPlex, Tecan, Männedorf, Switzerland) [70,71]. The results were derived using a calibration curve (range: 4.94 ng/mL–400 ng/mL) and are expressed as mean values ± SD (%) relative to the control (untreated cells) of five independent experiments performed in triplicate.

### 4.13. CD44 ELISA Kit

The presence of CD44 in Caco-2 and NHEK cell lysates was measured using an ELISA kit (Thermoscientific, Waltham, MA, USA) according to the manufacturer’s instructions [72]. Briefly, 100 μL of each diluted sample was added to a strip well and incubated at 37 °C for 2 h; then, the supernatants were removed, and 100 μL of Biotin-antibody was added to each of them and incubated for 1 h at 37 °C. When the time was over, the plate was washed three times with a wash buffer and then 100 μL of HRP-avidin was added to each well and incubated for 1 h; after five washes, 90 μL of TMB Substrate was added and incubated at 37 °C in the dark. After 15–30 min, 50 μL of Stop Solution was put in each well, and the plate was read immediately at 450 nm using a spectrometer (Infinite 200 ProMPlex, Tecan, Männedorf, Switzerland). The results were obtained by comparing the data to the standard curve (0.13 to 4 ng/mL). They are expressed as a percentage (mean ± SD) relative to the control (0 line) of five independent experiments performed in triplicate.

### 4.14. Crystal Violet

After each treatment, NHEK cells were washed and stained with 100 μL of 0.1% crystal violet in water (Merck Life Science, Rome, Italy) for 20 min at room temperature and then fixed with 1% glutaraldehyde for 15 min at room temperature. Then, 100 μL of 10 acetic acid was added to the multi-well plate, and after mixing, the absorbance at 595 nm was measured with a spectrophotometer (Infinite 200 Pro MPlex, Tecan, Männedorf, Switzerland). The estimated number was derived by comparing the results to control cells (control T0), which were inspected before stimulation, and the change in control (untreated) cells was also reported every 24 h [3]. The results are reported as the mean ± SD (%) compared to the control (untreated samples) of five independent experiments performed in triplicate.

### 4.15. MMP9 ELISA Kit

The MMP9 protein level was measured using an ELISA kit (Abcam, Cambridge, UK) that detects the presence of MMP9 protein in NHEK cell lysates, as directed by the manufacturer [3]. The samples were read immediately at 450 nm using a spectrometer (Infinite 200 ProMPlex, Tecan, Männedorf, Switzerland). The results were obtained by comparing the data to the standard curve (range: 105.47 pg/mL–6750 pg/mL) and are expressed as a percentage (mean ± SD) relative to the control (0 line) of five independent experiments performed in triplicate.

### 4.16. Ki67 ELISA Kit

Ki67 expression was measured using an ELISA kit (Abcam, San Diego, CA, USA) that detects the expression of Ki67 in NHEK cell lysates, as directed by the manufacturer [56]. The samples were read immediately at 450 nm using a spectrometer (Infinite 200 ProMPlex, Tecan, Männedorf, Switzerland). The results were obtained by comparing the data to the standard curve (ranging from 31.25 pg/mL to 2000 pg/mL). They are expressed as a percentage (%) relative to the control (0 line) of five independent experiments performed in triplicate.

### 4.17. Cell Migration Assay

The cell migration test was performed as described in the literature [73]. Before the cells were seeded, 60 mm Petri dishes were coated with non-animal HA at the concentration chosen. Cells were seeded only on the left side of the plate, with a holder positioned at an angle of about 10°; after 4 h (T0), the holder was removed, and KBM media was added. A phase-contrast microscope (Leica, Wetzlar, Germany) was used to detect cell migration every 24 h for 144 h. Migration was determined for each period using the ImageJ (ImageJ Version 1.32J., Sun-Java version 1.54k) image processing tool. The results are reported as the mean ± SD (%) of migrated cells compared to the control (untreated samples) of five independent experiments performed in triplicate.

### 4.18. Wound Healing Test

The wound closure (wound healing) test was carried out exactly as stated in the literature [3]. An incision was produced in the cell monolayer with a sterile p200 tip to simulate a wound. Afterwards, the cells were stimulated with non-animal HA after 4 h (time zero) and monitored every 24 h for 144 h. A phase-contrast microscope (Leica, Wetzlar, Germany) was used to study the repopulation of injured areas. The size of the denuded area was established by analysing digital photographs acquired in six distinct areas with the ImageJ image processing application. The results are expressed as the mean ± SD (%) of migrating cells compared to the control.

### 4.19. Western Blot Analysis

At the end of each stimulation, keratinocytes were rinsed with cold PBS 1× (Merck Life Science, Rome, Italy) and lysed using a Complete Tablet Buffer (Roche, Basel, Switzerland) enriched with 2 mM sodium orthovanadate (Na_3_VO_4_), 1 mM phenylmethanesulfonylfluoride (PMSF) (Merck Life Science, Rome, Italy), a 1:50 phosphatase inhibitor mix (Merck Life Science, Rome, Italy), and a 1:200 protease inhibitor mix (Merck Life Science, Rome, Italy) to yield a total protein extract that underwent laboratory analysis which was centrifuged at 14,000× *g* for 20 min at 4 °C. Subsequently, 40 µg of protein per extract was applied to 8% and 10% SDS-PAGE gels and transferred onto a polyvinylidene difluoride (PVDF) membrane, which was incubated overnight with specific primary antibodies such as mAChR M1 (1:500, Santa Cruz, CA, USA), mAChR M3 (1:500, Santa Cruz, CA, USA), and mAChR M5 (1:500, Santa Cruz, CA, USA). The expression levels of all proteins were normalised and confirmed by detecting β-actin (1:5000, Merck Life Science, Rome, Italy) and are presented as the mean ± SD (%) relative to the control value (line 0).

### 4.20. Statistical Analysis

Collected data were processed with Prism GraphPad 9.4.1 statistical software using one-way analysis of variance (ANOVA) followed by the Bonferroni post-hoc test to verify the assumption. Comparisons between two groups were performed using a two-tailed Student’s *t*-test. Multiple group comparisons were analysed using a two-way ANOVA followed by a two-tailed Dunnett post-hoc test to verify the assumption. All results are expressed as the mean ± SD of at least five independent experiments performed in triplicate. Differences with *p* < 0.05 were considered statistically significant.

## 5. Conclusions

In conclusion, our findings, for the first time, lend support to the hypothesis regarding the potential oral administration of high-molecular-weight hyaluronic acid, referred to as non-animal HA 0.01%, in conjunction with the probiotic strain *Bifidobacterium longum* novaBLG1 10 mg/mL to maintain skin health. This combination exhibits excellent intestinal tolerance, ensuring that the biological effects of hyaluronic acid are preserved and promoting its transport to the skin for maximum effectiveness. Furthermore, the survey clarified the dosage and frequency for the administration of this combination, suggesting a regimen of administration every 48 h throughout an extended 144 h treatment duration. Nevertheless, further comprehensive studies are necessary to validate our results.

## Figures and Tables

**Table 1:** Permeability values of the agents selected for the intestinal in vitro model. Data < 0.2 × 10^−6^ cm/s indicate inadequate absorption with bioavailability < 1%, data between 0.2 and 2 × 10^−6^ cm/s indicate 1 to 90% bioavailability, and data > 2 × 10^−6^ cm/s show over 90% bioavailability. Combination = non-animal HA 0.01% + *Bifidobacterium longum* novaBLG1 10 mg/mL. α *p* < 0.05 vs. sodium hyaluronate 0.01%; β *p* < 0.05 vs. non-animal HA 0.01%.

	Time Hours (h)	2 h	3 h	4 h	5 h	6 h
Substances						
Sodium hyaluronate 0.01%		0.28 × 10^−6^	0.33 × 10^−6^	0.36 × 10^−6^	0.35 × 10^−6^	0.30 × 10^−6^
^α^ Non-animal HA 0.01%		1.32 × 10^−6^	1.43 × 10^−6^	1.67 × 10^−6^	1.37 × 10^−6^	1.22 × 10^−6^
^αβ^ Combination		1.43 × 10^−6^	1.61 × 10^−6^	1.81 × 10^−6^	1.95 × 10^−6^	1.58 × 10^−6^

## Data Availability

The Laboratory of Physiology (F. Uberti) collects raw data and takes appropriate procedures to preserve them in a secure system forever. The corresponding author can provide this study’s data upon reasonable request.

## Abbreviations

Adv DMEM	Advanced Dulbecco’s Modified Eagle’s Medium
Adv DMEM-F12	Advanced Dulbecco’s Modified Eagle’s Medium/Nutrient F-12 Ham’s
APQ3	aquaporin-3
CD44	cluster of differentiation 44
cfu	colony formant unit
ECM	extracellular matrix
ELISA	Enzyme-Linked Immunosorbent Assay
EMA	European Medicines Agency
FBS	foetal bovine serum
FDA	Food and Drug Administration
HA	hyaluronic acid
HMW-HA	high-molecular-weight hyaluronic acid
KBM	keratinocyte basal medium
KGM2	keratinocyte growth supplements
Ki67	Kiel 67 protein
LMW-HA	low-molecular-weight HA
mAChR M1	muscarinic acetylcholine receptor type 1
mAChR M3	muscarinic acetylcholine receptor type 3
mAChR M5	muscarinic acetylcholine receptor type 5
MMP9	matrix metalloproteinase-9
MTT	3-(4,5-Dimethylthiazol-2-yl)-2,5-diphenyltetrazolium bromide
Papp	apparent permeability coefficient
PBS	phosphate-buffered saline
PMSF	phenylmethanesulfonylfluoride
PVDF	polyvinylidene difluoride
ROS	reactive oxygen species
TEER	trans-epithelial electrical resistance
TJ	tight junction
TLR4	Toll-like receptor 4
TNFα	tumour necrosis factor alpha
ZO-1	Zonula occludens-1
